# The Prognostic Significance of miR-21 Expression among Surgically Resected Hepatocellular Carcinoma Patients: Evidence from a Meta-Analysis and Retrospective Cohort Study

**DOI:** 10.1155/2020/8848158

**Published:** 2020-12-21

**Authors:** Xujian Huang, Yongfu Xiong, Jialin Yang, Gang Yang, Jingdong Li

**Affiliations:** ^1^Department of Hepatobiliary Surgery, Affiliated Hospital of North Sichuan Medical College, Nanchong 637000, China; ^2^Institute of Hepato-Biliary-Pancreatic-Intestinal Disease, North Sichuan Medical College, Nanchong 637000, China; ^3^Medical Imaging Center, Nanchong Central Hospital/Second School of Clinical Medicine, North Sichuan Medical College, Nanchong 637000, China

## Abstract

**Background:**

To date, microRNA-21 (miR-21) has been reported to be associated with the prognosis of hepatocellular carcinoma (HCC) in various studies, yet the results were inconsistent. The purpose of this two-part study, consisting of a retrospective cohort study and a meta-analysis, sets out to determine the prognostic role of miR-21 expression among HCC patients who underwent surgical resection*. Methods*. In this study, we first detected miR-21 expression in HCC patients by quantitative real-time PCR (qRT-PCR). Patients were divided into a high miR-21 expression group and a low miR-21 expression group according to the median level of miR-21 expression in tumor tissues. The survival outcomes of the two groups were analyzed by the Kaplan-Meier method with the log-rank test. Multivariate analysis of the prognostic factors was performed with the Cox regression model. Subsequently, eligible studies were obtained by searching on PubMed, Cochrane Library, and Web of Science, and a meta-analysis was performed to assess the prognostic role of miR-21 expression among HCC.

**Results:**

The qRT-PCR analysis results of our cohort study showed miR-21 expression was significantly upregulated in HCC tissues when compared with adjacent nontumor tissues. Multivariate analysis suggested that miR-21 expression was an independent prognostic factor for overall survival (OS) (hazard ratio, HR = 2.361) and disease-free survival (DFS) (HR = 2.024) in HCC patients who underwent surgical resection. A total of 10 studies with 969 patients were enrolled in the meta-analysis, consisting of 9 studies from the database search and our cohort study. We observed that elevated miR-21 expression can predict poor OS (HR = 2.24, 95%CI = 1.73‐2.91, *P* < 0.001) and DFS/recurrence-free survival (RFS) (HR = 2.44, 95%CI = 1.62‐3.67, *P* < 0.001) in surgically resected HCC patients.

**Conclusions:**

Our study demonstrated that miR-21 high expression among surgically resected HCC patients is a prognostic factor that indicated adverse survival.

## 1. Introduction

Liver cancer is one of the most common cancers in the world, results in 841,000 new cases per year, and kills 782,000 people annually [1, 2]. Hepatocellular carcinoma (HCC) is the most frequent type, accounting for 75%-85% of all primary liver cancers, and is the third leading cause of malignant tumor-related deaths, with increasing incidence each year [3]. Radical resection is the main method for the treatment of liver cancer [4]. In recent years, with the accumulation of surgical experience, the application of advanced instruments, and the progress of perioperative evaluation and management, the 5-year survival rate after hepatectomy has increased, but the oncology prognosis of patients with HCC is still unfavorable because of its biological characteristics such as rapid invasive growth, poor differentiation, and early metastasis [5–7]. Therefore, it is crucial to identify prognostic markers which can identify different phenotypes with differences in clinical characteristics and prognosis of HCC for guiding clinical management.

MicroRNAs (miRNAs) are a large family of small, approximately 21-nucleotide-long, noncoding RNAs that are predicted to control the activity of approximately 30% of all protein-coding genes and have been shown to participate in the regulation of almost every cellular process in mammals [8, 9]. More importantly, recent studies suggest that microRNAs also play a significant role in carcinogenesis acting either as oncogenes or tumor suppressors [10, 11]. miR-21 is one of the most frequently observed cancer-related microRNAs [9, 12, 13]. Previous studies have observed that miR-21 elevates in many cancers such as breast cancer, lung cancer, pancreatic cancer, stomach cancer, and hepatocellular carcinomas and acts as a key factor mediating the growth, development, and progression of tumors [10, 14–16]. Recent reports have suggested that miR-21 expression is related to survival in HCC patients [12, 13, 17, 18]; however, the evidence remains inconclusive. Thus, we conducted a two-part study comprising a retrospective cohort study and a meta-analysis to further explore the prognostic significance of miR-21 expression in HCC patients who underwent surgical resection.

## 2. Materials and Methods

### 2.1. Study Population

This study was approved by the Research Ethics Committee of the Affiliated Hospital of North Sichuan Medical College, and informed consent was provided by all 166 patients with HCC who underwent radical hepatectomy from January 2013 to December 2015. According to the median level of miR-21 expression in cancer tissues, these patients were divided into a high miR-21 expression group and a low miR-21 expression group. The diagnosis of HCC was confirmed by clinical resection and pathology. Patients who received neoadjuvant chemotherapy, patients with distant metastases during surgery, and patients with other malignancies were excluded from this study. The operation was performed by several surgeons according to the standard criteria for hepatectomy in our hospital.

### 2.2. Histopathological Evaluation

**Figure 1:**
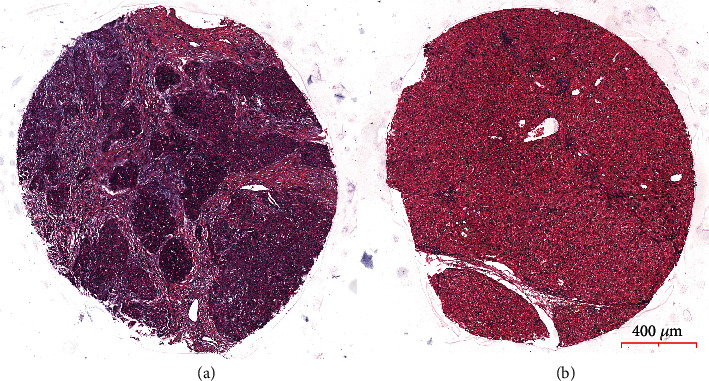
H&E staining of HCC specimen (a) and adjacent nontumor tissue (b).

Histopathological evaluation of all surgical specimens was fulfilled based on standard path procedures, and hematoxylin and eosin- (H&E-) stained slides from all cases were reviewed by hepatic pathologists without any knowledge of the clinical outcomes (Figure 1).

### 2.3. RNA Isolation and qRT-PCR

Total RNA was extracted from the surgically resected tissues by using the TRIzol RNA extraction kit. cDNA was generated using the PrimeScript RT reagent kit (Takara) in a 20 *μ*l final reaction volume containing 0.5 *μ*g of RNA, 0.5 *μ*l PrimeScript RT enzyme mix, 4 *μ*l 5x PrimeScript buffer, and 1 *μ*l RT primer and incubated at 42°C for 60 min and at 85°C for 5 min. The quantitative real-time PCR assay was performed to evaluate miR-21 expression using SYBR Premix Ex Taq (Takara) and measured in a LightCycler 480 System (Roche). The amplification profile was denatured at 95°C for 10 min, followed by 45 cycles of denaturation at 95°C for 15 s, annealing at 60°C for 30 s, and extension at 72°C for 1 min. The relative expression of miR-21 was calculated and normalized using the 2^-*ΔΔ*Ct^ method relative to U6 small nuclear RNA.

## 3. Statistical Analysis

Using SPSS25.0 statistical software (SPSS Inc., Chicago, USA) to evaluate the prognostic significance of miR-21. Relationships between miR-21 expression and patient characteristics were investigated using Pearson's *χ*^2^ test and Spearman's correlation analysis. The differences in overall survival (OS) and disease-free survival (DFS) between the two groups were analyzed using the Kaplan-Meier method with the log-rank test. Univariate analysis was used to establish the potential prognostic factors for OS and DFS, and multivariate analysis for significant factors was performed by Cox proportional hazard regression models. A *P* value of *<*0.05 indicated a statistically significant difference.

## 4. Meta-Analysis

This meta-analysis was performed in accordance with the guidelines of the Preferred Reporting Items for Systematic Reviews and Meta-Analyses (PRISMA) [19].

### 4.1. Search Strategy

We performed a systematic literature search on PubMed, Cochrane Library, and Web of Science databases for articles that assessed the relationship between miRNA-21 and the prognosis of HCC patients who underwent radical resection until December 31, 2019, and with no lower date limit. The following terms were used: (1) “microRNA-21” or “miR-21” or miRNA-21, and (2) “carcinoma∗, hepatocellular” or “hepatocellular carcinoma∗” or “hepatoma∗” or “liver cell carcinoma∗” or carcinoma∗, liver cell, and (3) “prognos∗” or “survival” or “outcome∗”.

### 4.2. Selection Criteria

**Figure 2:**
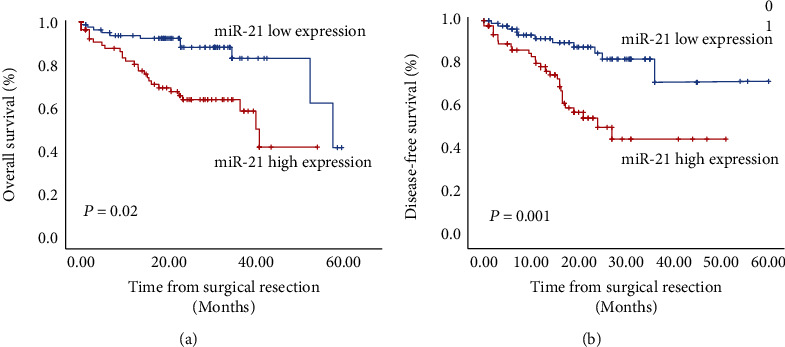
Kaplan-Meier curves for overall survival (a) and disease-free survival (b) according to miR-21 expression in HCC patients who underwent surgical resection.

All of the eligible studies met the following criteria: (i) studied patients with HCC based on histopathological confirmation, (ii) studied patients with HCC underwent surgical resection, and (iii) investigated the survival outcome or the correlation between miR-21 expression and the clinical characteristics. Studies were excluded based on any of the following criteria: (i) reviews, letters, and case reports; (ii) non-English studies; (iii) studies had overlapping or duplicate data; and (iv) lacked key information for calculation with methods established by Parmar et al., Williamson et al., and Tierney et al. [20–22]. A flow diagram of the study selection process is summarized in Figure 2.

### 4.3. Quality Assessment

The Newcastle–Ottawa Scale [23] was used to evaluate the quality of each study included in the meta-analysis and three aspects were generally assessed: population selection, study comparability, and reporting of the outcome, with a score ranging from 0 to 9. A study with a score of ≥6 was considered to be of high quality.

### 4.4. Data Extraction

Two investigators (Huang Xujian and Yang Jialin) independently reviewed each eligible study and extracted data from studies following the guidelines before mentioned selection criteria [24]. The controversial issues were resolved by discussion, and consensus was reached by the third investigator (Li Jingdong) when there were disagreements between two investigators. The following details were summarized from each eligible study: first author's name, published year, country, ethnicity, sample size, patients' sex, detection method, cutoff value, HRs with 95% confidence intervals (CIs), and *P* value of miR-21 for OS and DFS/RFS. If only survival curves were available, data were extracted using the method described by Tierney et al. [21].

### 4.5. Statistical Methods

Pooled hazard ratio (HR) with 95% CI was used to evaluate the relation between high miR-21 expression and prognosis of HCC patients. Pooled HR > 1 implied unfavorable prognosis for the groups with elevated miR-21 expression and had statistical significance if the 95% CI did not overlap 1, while HR < 1 implied a favorable prognosis. The heterogeneity was assessed using *I*^2^ statistic (*P* < 0.1 or *I*^2^ > 50% indicate significant heterogeneity) described by Higgins et al. [25]. The random-effects model was used if significant heterogeneity exists among studies; otherwise, the fixed-effects model was used. Publication bias was assessed using the funnel plot. Forrest plots were used to estimate the effect of miR-21 expression on survival outcomes (OS and DFS/RFS). Data analyses were performed using RevMan software, version 5.3 (the Nordic Cochrane Centre, Cochrane Collaboration, Copenhagen, Denmark) and Stata 14.0 (Stata Corporation, College Station, TX, USA). *P* < 0.05 denoted statistical significance.

## 5. Results

### 5.1. The Expression of miR-21 Is Significantly Upregulated in Human HCC Tissues

**Figure 3:**
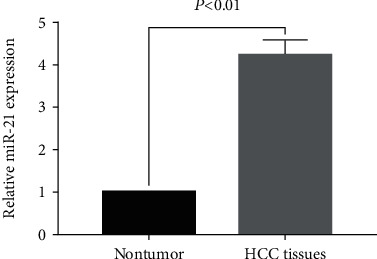
Relative expression levels of miR-21 in 166 paired HCC and adjacent nontumor tissues.

The qRT-PCR assay was performed to determine the expression level of miR-21 in 166 cases of HCC and their matched nontumor tissues. The results showed that the expression of miR-21 in hepatocellular carcinoma was significantly higher than that in nontumor tissues (*P* < 0.05, Figure 3). Based on the median level of miR-21 in cancer tissues, 166 HCC patients were divided into a high miR-21 expression group (*n* = 83) and a low miR-21 expression group (*n* = 83) (Table 1).

### 5.2. Association of miR-21 Expression with Clinic-Pathological Characteristics of HCC Patients

For a better understanding of the clinical significance of miR-21 expression in HCC, we further investigated the clinic-pathological characteristics of HCC. As shown in Table 1, the high expression level of miR-21 was closely related to tumor size, tumor number, tumor differentiation, and TNM stage (*P* < 0.05). Nevertheless, there were no associations between the expression of miR-21 and the other clinic-pathological parameters including age, sex, vascular infiltration, *α*-fetoprotein (AFP) level, and cirrhosis (*P* > 0.05) (Table 1).

### 5.3. The Impact of miR-21 Expression on OS in HCC

To determine whether the miR-21 expression level is a significant predictor for OS after hepatectomy, the Kaplan-Meier OS curve of the HCC patients according to the status of the miR-21 level was examined. The OS of patients with high miR-21 expression was significantly lower than those with low miR-21 expression (*P* = 0.02; Figure 2(a)). Univariate analysis showed that tumor size, tumor number, tumor differentiation, vascular infiltration, cirrhosis, TNM stage, and high miR-21 expression were related to a poor overall survival for patients with HCC. Further multivariate analysis showed that high miR-21 expression (HR = 2.361; 95%CI = 1.016‐5.490; *P* = 0.046), vascular infiltration (HR = 2.56; 95%CI = 1.162‐5.639; *P* = 0.019), and tumor size (HR = 3.902; 95%CI = 1.273‐11.956; *P* = 0.017) were independent prognostic factors for OS. The results are shown in Table 2.

### 5.4. The Impact of miR-21 Expression on DFS in HCC

Kaplan-Meier analysis demonstrated that DFS was significantly shorter in the miR-21 high expression group compared with the miR-21 low expression group (*P* = 0.001, Figure 2(b)). In addition, univariate Cox regression analysis indicated that miR-21 expression, tumor number, tumor differentiation, vascular infiltration, cirrhosis, and TNM stage were closely associated with DFS (Table 3). However, multivariate Cox analysis suggested that only miR-21 expression and tumor number were independent predictors of DFS in patients with HCC (Table 3).

### 5.5. Identification and Eligibility of Relevant Studies in the Meta-Analysis

**Figure 4:**
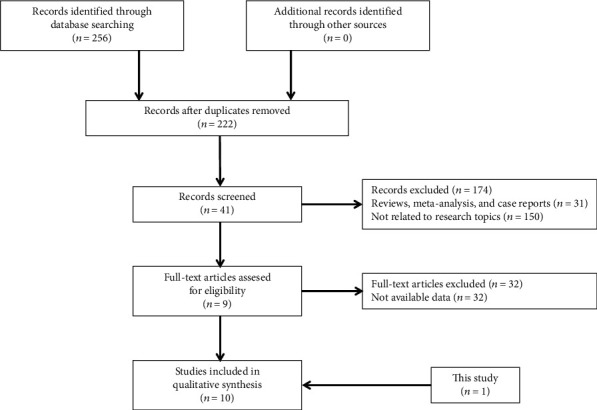
Flow chart of the study selection process.

A flow diagram of the study selection process is summarized in Figure 4. Finally, 9 previously published articles and our current cohort study were included in the meta-analysis [17, 18, 26–32]. These eligible studies were published between 2012 and 2019 and included a total of 969 patients with HCC from China, Japan, South Korea, and Greece. These eligible studies were all retrospective cohort studies. 10 studies investigated the relation of miR-21 with overall survival (OS) of HCC patients, and 6 explored its connection with disease-free survival (DFS). The method of miR-21 expression detection was all quantitative real-time polymerase chain reaction (qRT-PCR). Characteristics of the eligible studies are summarized in Table 4.

### 5.6. Meta-Analysis of miR-21 Expression and OS in HCC Patients

**Figure 5:**
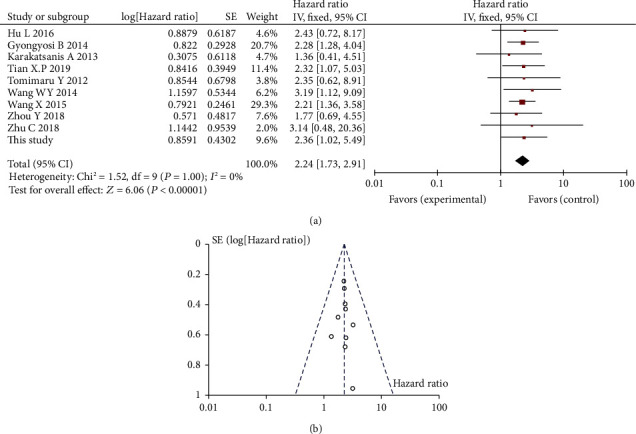
Forest plot (a) and funnel plot (b) for the association between miR-21 expressions with overall survival (OS).

Figure 5 shows the forest plot for the survival data. The combined HR was 2.24 (95%CI = 1.73‐2.91, *P* < 0.001), indicating that elevated miR-21 expression was significantly predictive of poor OS in HCC patients undergoing surgical resection. The fixed model effect was used to synthesize the data, since no significant heterogeneity existed among these studies (*I*^2^ = 0%, *P* = 1.0).

### 5.7. Meta-Analysis of miR-21 Expression and DFS/RFS in HCC Patients

Among the eligible studies, 6 referred to the correlation between miR-21 expression and DFS/RFS, including 5 previously published studies and our cohort study. Since recurrence-free survival (RFS) and disease-free survival (DFS) are indicators of disease progression, such as locoregional recurrence or distant metastasis, we merged them for a pooled analysis. Considering that there was no significant heterogeneity between studies (*I*^2^ = 0%, *P* = 0.62), we chose the fixed-effects model to perform the meta-analysis. Forest plots of the meta-analyses for miR-21 expression are depicted in Figure 5. Combined data from eight studies showed that HCC patients with high miR-21 expression had shorter DFS/RFS, with a pooled HR estimate of 2.44 (95%CI = 1.62‐3.67, *P* < 0.001).

### 5.8. Publication Bias and Sensitivity Analysis

**Figure 6:**
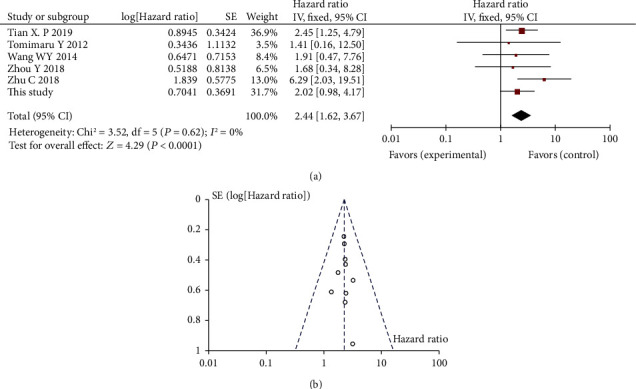
Forest plot (a) and funnel plot (b) for the association between miR-21 expression with recurrence-free survival (RFS)/disease-free survival (DFS).

The funnel plot test was used to evaluate publication bias. As shown in Figures 5 and 6, the funnel plots were almost symmetric in OS studies, as well as in DFS/RFS studies. Sensitivity analysis was performed by eliminating the highest weighted study, and there was no individual study that substantially changes the overall HR, which indicates the reliability of our results.

## 6. Discussion

Since the initial recognition of the relation between miR-21 and cancer in 2005 [33], miR-21 has gained wide concern in cancer research for its crucial role in regulating the expression of oncogenes and tumor suppressors. Recently, a series of quantitative analyses have been performed to identify the prognostic role of miR-21 in various cancers. Zhu et al. demonstrated that elevated miR-21 moderately predicts poor overall survival (OS) in general carcinomas (HR = 1.903, 95%CI = 1.713‐2.113, *P* < 0.001), especially pancreatic cancer patients [34]. Similar results were summarized in Hu et al.'s analyses, with pooled HR for OS 2.05 (95%CI = 1.71–2.46, *P* < 0.001) [35]. In gastric cancer, Wang et al. reported higher miR-21 expression could significantly predict poorer survival with pooled HR for OS 2.00 (95%CI = 1.39‐2.88, *P* < 0.01) [36]. However, there were also insignificant or opposite results in some studies. In a meta-analysis of 1,163 non-small-cell lung cancer (NSCLC) cases, Ma et al. indicated the HR for OS is 2.19 (95%CI≔0.76–6.30, *P* = 0.15), which means the miR-21 expression has limited prognostic significance on NSCLC [37]. In general, the prognostic role of miR-21 in cancers is still disputable.

Similarly, different results have been found in studies related to the expression of miR-21 in HCC and its prognostic value. Tian and his colleagues found that the high expression of miR-21 was negatively correlated with the prognosis of patients with HCC after surgery [28]. In another study, Gyongyosi et al. reported that in 20 HCC patients who received sorafenib treatment, the high expression of miR-21 was not an independent predictor of OS [26]. Why is the prognostic significance of miR-21 in HCC patients controversial? First, the treatment of HCC is characterized by the coexistence of multidisciplines and multitreatments, including hepatectomy, liver transplantation, and transarterial chemoembolization (TACE), and different treatments may lead to these differences [6, 38, 39]. Secondly, factors such as region-specific, race-specific, gender-specific, or age-specific factors may affect prognostic outcomes and produce statistical heterogeneity.

Surgical resection is a recommended treatment option in HCC patients with resectable tumor [4, 38]. Hence, we carried out this two-step study, which focused on thoroughly exploring the relationship between the expression of miR-21 and the prognosis of HCC patients who underwent surgical resection. Our current cohort study showed that miR-21 was overexpressed in HCC tissues compared with adjacent normal tissues, and high expression of miR-21 was significantly correlated with shorter OS and DFS/RFS, which was consistent with the subsequent meta-analysis results. In addition, we also evaluated the relationship between the expression of miR-21 and the clinicopathological features of patients with HCC. Our results showed the high expression level of miR-21 was closely related to tumor size, tumor number, tumor differentiation, and TNM stage, suggesting that the expression of miR-21 may be related to the malignant characteristics of the tumor. Our results further clarify the close relationship between the overexpression of miR-21 and the progression, metastasis, and death of HCC.

To our knowledge, this two-part study is the first meta-analysis to thoroughly assess the relationship between miR-21 expression and the prognosis of HCC patients that underwent surgical resection. However, it did have several limitations. First, all eligible studies in the meta-analysis were of a retrospective nature, which might have introduced a degree of bias. Second, the cutoff values were different in various studies. Indeed, there is no common threshold value to define miR-21-positive expression in patients with HCC. Third, when the individual HR together with its variance were not reported in the selected articles, the data are extracted from the survival curve using the method described by Tierney et al. [21]. The estimated HR may be less reliable than the one obtained directly from published statistics.

## 7. Conclusion

Our retrospective cohort study and meta-analysis demonstrated that miR-21 high expression among surgically resected HCC patients is a prognostic factor that indicated adverse survival.

## Figures and Tables

**Table 1:** Correlation between miR-21 expression and clinicopathological features.

Patient characteristics	Number	miR-21 expression		*P* value
		Low (*N* = 83)	High (*N* = 83)	
Gender				
Male	87	48	39	0.162
Female	79	35	44	
Age (years)				
<55	46	25	21	0.488
≥55	120	58	62	
Tumor size (cm)				
≤5	132	74	58	0.002
>5	34	9	25	
Tumor number				
Single	133	72	61	0.032
Multiple	33	11	22	
Serum AFP (*μ*g/l)				
<400	47	21	26	0.389
≥400	119	62	57	
Liver cirrhosis				
Absence	38	17	21	0.46
Presence	128	66	62	
Tumor differentiation				
Well+moderate	101	63	38	<0.001
Poor	65	20	45	
TNM stage				
I-II	96	60	36	<0.001
III-IV	70	23	47	
Vascular infiltration				
Absence	121	66	55	0.055
Presence	45	17	28	

miR-21: microRNA-21; AFP: *α*-fetoprotein.

**Table 2:** Univariate and multivariate Cox regression analysis for OS.

Variables	Univariate analysis		Multivariate analysis	
	HR (95% CI)	*P* value	HR (95% CI)	*P* value
Age (years)	1.160 (0.556-2.418)	0.693		
Gender	0.781 (0.412-1.481)	0.45		
Tumor size	3.784 (1.339-10.700)	0.012	3.902 (1.273-11.956)	0.017
Tumor number	2.377 (1.217-4.640)	0.011		
Tumor differentiation	2.766 (1.427-5.361)	0.003		
TNM stage	3.232 (1.627-6.420)	0.001		
Vascular infiltration	2.467 (1.273-4.784)	0.007	2.56 (1.162, 5.6399)	0.019
Cirrhosis	1.971 (1.005-3.866)	0.048		
AFP	1.731 (0.855-3.505)	0.127		
miR-21 expression	3.368 (1.622-6.993)	0.001	2.361 (1.016-5.490)	0.046

OS: overall survival; miR-21: microRNA-21; AFP: *α*-fetoprotein; HR: hazard ratio; CI: confidence interval.

**Table 3:** Univariate and multivariate Cox regression analysis for DFS/RFS.

Variables	Univariate analysis		Multivariate analysis	
	HR (95% CI)	*P* value	HR (95% CI)	*P* value
Age (years)	1.201 (0.582-2.478)	0.62		
Gender	0.760 (0.402-1.437)	0.398		
Tumor size	1.773 (0.879-3.575)	0.11		
Tumor number	2.377 (1.082-8.667)	0.035	2.243 (1.035-4.862)	0.041
Tumor differentiation	2.994 (1.540-5.823)	0.001		
TNM stage	3.381 (1.697-6.733)	0.001		
Vascular infiltration	2.053 (1.078-3.910)	0.029		
Cirrhosis	2.009 (0.374-10.791)	0.416		
AFP	1.845 (1.022-3.329)	0.042		
miR-21 expression	3.179 (1.569-6.442)	0.001	2.02 (1.042-3.92)	0.037

DFS/RFS: disease-free survival/recurrence-free survival; miR-21: microRNA-21; AFP: *α*-fetoprotein; HR: hazard ratio; CI: confidence interval.

**Table 4:** The characteristics of the pooled studies.

Study (Ref.)	Country	Ethnicity	Sample size	Method	Cutoff	Results	Score (NOS)	Ref.
Tomimaru Y 2012	Japan	Asian	126	qRT-PCR	Mean: 0.754	OS/RFS	8	[28]
Karakatsanis A 2013	Greece	Caucasian	60	qRT-PCR	3.07-fold of control	OS	7	[27]
Gyongyosi B 2014	Italy	Caucasian	20	qRT-PCR	Median	OS	6	[25]
Wang WY 2014	China	Asian	119	qRT-PCR	Median	OS/DFS	8	[29]
Wang X 2015	China	Asian	97	qRT-PCR	Median	OS	7	[30]
Hu L 2016	China	Asian	32	qRT-PCR	Median	OS	6	[26]
Zhu C 2018	China	Asian	50	qRT-PCR	Median	OS/DFS	8	[16]
Zhou Y 2018	China	Asian	83	qRT-PCR	Score ≥ 2	OS/DFS	6	[31]
Tian X.P 2019	China	Asian	124	qRT-PCR	Median	OS/RFS	8	[18]
Our study	China	Asian	166	qRT-PCR	Median	OS/DFS	7	
